# Validation of the use of POSSUM score in enteric perforation peritonitis - results of a prospective study

**DOI:** 10.4314/pamj.v9i1.71197

**Published:** 2011-06-23

**Authors:** Sunil Kumar, Amit Gupta, Sujata Chaudhary, Neeraj Agrawal

**Affiliations:** 1 Department of Surgery, University College of Medical Sciences and associated Guru Teg Bahadur Hospital, New Delhi-110095, India; 2 Department of Anaesthesia, University College of Medical Sciences and associated Guru Teg Bahadur Hospital, New Delhi-110095, India

**Keywords:** Enteric perforation, Peritonitis, POSSUM, P-POSSUM

## Abstract

**Introduction:**

The objective of the study was to present our last 5-years experience of peritonitis and validate POSSUM score in predicting mortality and morbidity in patients of enteric perforation (EP) peritonitis.

**Methods:**

Data was collected prospectively for all peritonitis cases admitted in single surgical unit from January 2005 to December 2009. Parameters for calculating POSSUM were also retrieved; in these patients, O:E (Observed vs. Expected) ratio of mortality and morbidity were estimated after calculating predicted mortality and morbidity by exponential regression equations.

**Results:**

887 patients with peritonitis were admitted and treated in this unit during the 5 years of study period. Duodenal (n=431; 48%) followed by ileal (n=380; 42.8%) perforations were the commonest. Mean age of the patients was 34 years and 86% were males. Mean delay in presentation was 78.5 hrs. Mean duration of hospital and ICU stay was 13 and 7.2 days. Postoperative complications were seen in 481 (54%) patients, and 90 (10%) patients died. POSSUM scores and predicted mortality/morbidity were calculated in 380 patients of ileal perforation peritonitis; O:E ratio of mortality and morbidity were 0.47 and 0.85 in these patients.

**Conclusion:**

POSSUM and P-POSSUM are accurate tools for predicting morbidity and mortality respectively in EP patients. Though they may sometime over or under predict morbidity as well as mortality.

## Introduction

Typhoid fever is the commonest cause of ileal perforation in India. After initial resuscitation with intravenous fluids and correction of electrolyte imbalance, emergency laparotomy is performed to either repair the perforation or exteriorization of the bowel segment bearing the perforation.

The risk of postoperative morbidity and mortality can be predicted using several scoring systems. These scoring systems can also be used for surgical audits and qualitative assessments of different surgeons, hospitals and countries. POSSUM (Physiological and Operative Severity Score for the enumeration of Mortality and Morbidity) score, developed by Copeland et al in 1991, is one of the most widely used scoring system [1]. It uses 12 preoperative physiological and 6 operative variables on a four grade scoring system and the results are analysed using linear or exponential method of analysis; exponential method is better but more difficult. Since its advent, several modifications have come in existence to more accurately predict morbidity and mortality and also to include other subspecialties. It can also be used for risk adjustment in similar subgroups of patients in different settings so that outcome can be compared. Several studies have suggested it to be accurate. POSSUM (Physiological and Operative Severity Score for the enumeration of Mortality and morbidity) is the only scoring system which is meant for exclusive use in surgical cases. Of late, “Portsmouth” modification of POSSUM (P-POSSUM) scoring system has been introduced which predicts mortality more accurately than the former [2]. P-POSSUM (Portsmouth POSSUM) uses same variables but results are derived using simpler linear analysis method. A comparison of POSSUM and P-POSSUM scoring systems has never been done in patients with enteric perforation peritonitis.

The objective of this study was to validate the POSSUM score in predicting mortality and morbidity in enteric perforation peritonitis and to use risk adjustment with POSSUM to compare treatment groups in our set up.

## Methods

This prospective, non-randomized observational clinical study was conducted in single surgical unit at our institute from January 2005 to December 2009. 887 patients aged between 05 years to 75 years were treated for peritonitis during this period and were included in the study. Their demographic profile, detailed history and examination were recorded in predesigned proforma. Investigations for POSSUM scoring like haemoglobin, WBC count, blood urea, serum electrolytes, arterial blood gases, chest X-ray and ECG were done at admission. Investigations conducted to further confirm the diagnosis of typhoid, besides Widal test, included typhidot test, blood culture, urine culture, and stool culture wherever possible.

After blood sampling, intravenous antibiotics (Injection Ceftriaxone and metronidazole) were administered in all patients. All patients underwent correction of fluid and electrolyte imbalance through intravenous route, guided by urine output. Patients underwent laparotomy as soon as possible; operative findings were noted and tailored surgical procedure was carried out. All patients with ileal perforation underwent either ileostomy at the perforation site or repair of the perforation and proximal diverting ileostomy. Antibiotics were continued for 7 to 10 days. Intravenous fluids were continued for 3-4 days postoperatively. Oral fluids and feeds were started and tolerated, generally, on 3^rd^ or 4^th^ postoperative days. Patients were discharged on or around 10th postoperative days and then followed up in outpatient for a period of 6 months. Morbidity and mortality was recorded. Morbidity was treated as appropriate. Ileal perforations were considered to be of typhoid origin when patient was Widal positive (titre>1/80) with one or all of the following present: 1) Acute abdomen preceded by a variable period of high non-swinging fever, abdominal pain and toxaemia; 2) Typhidot positive according to manufacturer's instructions; 3) *S. typhi* isolated from blood, stool or urine cultures.

### Data analysis

Continuous data was presented as mean+SD. For calculation of significance between continuous variables between two separate groups, unpaired t- test was used. For calculation of significance between two proportions and percentages, Chi-square and Fischer's test were used.

### Methods of calculating POSSUM predicted morbidity or mortality [3]

POSSUM equation for morbidity:

Log_n_ R/(1-R) = -5.91 + (0.16 × physiological score) + (.19 × operative severity score)

POSSUM equation for mortality:

Log_n_ R/(1-R) = -7.04 + (.13 × physiological score) + (.16 × operative severity score)

### Methods of calculating number of patients to undergo morbidity or mortality

1) Linear analysis: the mean predicted risk (R) is multiplied with the total number of patients in group, to give the predicted number of patients; 2) Exponential analysis: it calculates the predicted number of individuals within each probability group by subtracting those with a risk more than the upper group limit from those with a risk more than lower group limit.

Using predicted and observed morbidity and mortality, O:E ratios were calculated. O:E ratio less than 1 meant over-prediction. O:E ratio more than one meant under-prediction. Observed/Expected (O:E) ratio were calculated and the difference between observed and expected mortality and morbidity was detected by chi-square test. P

Patients with ileal perforation underwent either ileostomy (subgroup A) or primary repair (subgroup B).These two subgroups were compared for POSSUM risk-adjusted morbidity and mortality to find the true status of morbidity and mortality in these two subgroups of patients. For this purpose, odds ratio for morbidity and mortality in patients in subgroup A was calculated against patients in subgroup B as reference. Then POSSUM morbidity and mortality scores were used for risk adjusting.

## Results

### General results (n=380)

The age of these patients ranged from 05 years to 75 years with mean of 32.07±14.24 years. There were 348 (91.8%) males. Physiological component of POSSUM score ranged from 12 to 34 with mean of 19.61 ±4.57 and operative score ranged from 11 to 19with mean of 17.82±1.89. Duodenum was the commonest site of perforation (Table 1). Surgical site infection (SSI) was the most common complication, found in 231(60.8%) patients. Other common complications were burst abdomen in 76 (20%), renal failure in 25(7.8%) and chest infection in 19 (5%). Hospital stay ranged from 5 days to 35 days, with mean of 13.73±6.7 days. Twelve patients received postoperative treatment in ICU at one or the other time during their hospital stay; mean±SD stay in ICU was 4.33±5.32 days. Eight patients expired (mortality-8.2%).

**Table 1:** Causes of peritonitis (n=887)

Causes of peritonitis	Number (%)
Peptic ulcer (duodenal+pyloric)	431 (48.5)
Ileal	380 (42.8)
Appendiceal	32(3.6)
Gastric*	18 (2.0)
Jejunal	8 (0.9)
Large intestinal	8 (0.9)
Pyoperitoneum (no perforation)	7 (0.7)
Gall bladder	3 (0.3)

* Five were malignant ulcers

### Relation between “at admission-complications” and duration of peritonitis (n=380)

**Figure 1:**
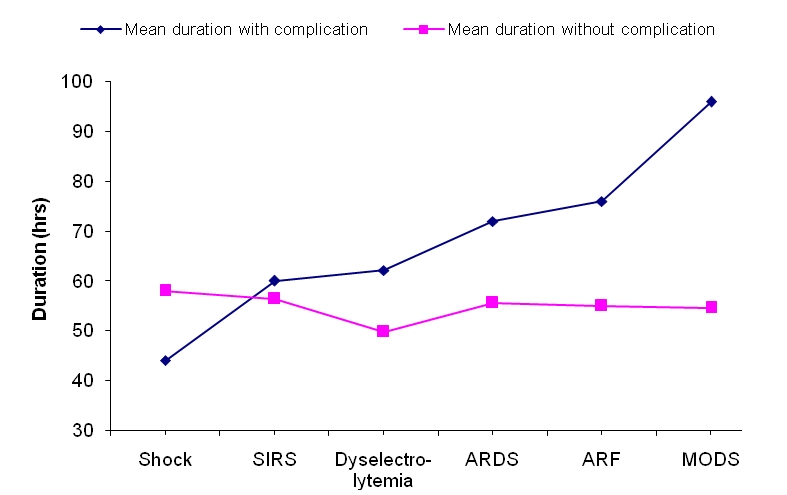
Relation between “at admission-complications” and duration of peritonitis

Close observation led us to recognize two distinct groups of patients at admission: one category of patients having one or more organ failure at admission, the other category of patients with apparently normal physiology (or no organ failure). The mean duration of peritonitis (in hours) in these two categories of patients was calculated and a graph was drawn (Figure 1). Mean duration of peritonitis in patients with no complications at the time of presentation was in the narrow band of 50-60 hrs. On the other hand, in patients who presented with complications duration of peritonitis ranged from 44 hrs to 96 hrs. This suggests that with increasing duration of peritonitis chances of patient presenting with complications like dyselectrolytemia, adult respiratory distress syndrome (ARDS), acute renal failure (ARF) and multi organ dysfunction syndrome (MODS) increases. However, shock at presentation showed an inverse relationship with duration of peritonitis i.e. shorter the duration of peritonitis, greater the chances of patient presenting with shock. The duration of peritonitis in patient presenting with or without systemic inflammatory response syndrome (SIRS) was comparable.

### Small bowel perforation: enteric versus Non-specific (n=53)

Ileum was the seat of perforation in 380 patients. Based on diagnostic criteria specified in the material and methods section, perforation of enteric origin (EP) was suspected in 327 (86.05%); remaining 24 (24.7%) patients were considered to have non-specific ileal perforation (NSIP). These two groups of patients were compared on several parameters in order to find if one could really differentiate. EP group of patients differed only in terms of higher BMI, tendency to present with higher (>2) systemic inflammatory response score (SIRS) and longer hospital stay than NSIP patients.

### Timing of perforation in EP patients (n=327)

Perforation occurred in first week of illness in most (n=17) EP patients. Ten patients presented with perforation in the second week of their illness. Remaining two had perforation in the third week of the illness.

### Value of culture, histopathology and typhidot in diagnosing EP (n=327)

Isolation of the bacteria is considered the gold standard against which sensitivity and specificity of another diagnostic test is measured. However, culture was negative in all of our patients (possibly due to indiscriminate use of antibiotics at family physician level. Histopathology (for erythrophagocytosis) and typhidot were positive in 19 and 20 cases, respectively.

### Value of POSSUM predicted morbidity for all patients (n=380)

POSSUM tended to over-predict morbidity, except in the lower risk bands. Overall O:E ratio was 0.85. The difference between observed and expected morbidity was statistically significant in exponential analysis (p=0.0219) (Table 2). P=0.0219 (significant)

**Table 2:** Exponential analysis for calculation of morbidity (n=380)

Risk %	Predicted morbidity	Observed morbidity	O/E
0-20	0	0	NA
20-40	16	12	0.75
40-60	92	68	0.74
60-80	124	112	0.90
80-100	52	48	0.92
**Total**	**284**	**240**	**0.85**

O/E: Observed/Expected

### Value of POSSUM predicted mortality for all patients (n=380)

Except in the middle risk bands (20-40), POSSUM tended to over predict mortality. Overall O:E ratio was 0.47. The difference between observed and expected mortality was statistically significant (p=0.0162) (Table 3).

**Table 3:** Exponential analysis for mortality using POSSUM (n=380)

Risk %	Predicted mortality	Observed mortality	O/E
0 – 10	4	0	0
10 – 20	32	8	0.25
20 – 30	20	16	0.8
30 – 40	8	8	1
40 – 50	4	0	0
>50	0	0	NA
**Total**	**68**	**32**	**0.47**

### Value of POSSUM predicted morbidity in EP patients (n=327)

POSSUM under predicted morbidity in EP patients especially in the low risk band. Overall O:E ratio was 1.27 and the difference between observed and predicted morbidity was not statistically significant (p value=0.0557).

### Value of POSSUM predicted mortality in EP patients (n=327)

POSSUM over predicted mortality in EP patients with O:E ratio of 0.4, but there was no statistical difference between predicted and observed mortality (p value=0.1403)

### Risk adjusted morbidity and mortality in patients undergoing ileostomy (subgroup A) versus patients undergoing primary repair (subgroup B)

Crude mortality rates were higher (10%) in subgroup A. Odds of morbidity and mortality were calculated in subgroup A against that in subgroup B as reference before and after risk adjustment. Odds of morbidity for subgroup A before risk adjustment was 0.625, with p value=0.704. When this odds ratio was adjusted with POSSUM morbidity scores, the odds of morbidity reduced to 0.068 and the p value was 0.115. This drop in odds ratio for morbidity was not significant. Odds of mortality for subgroup A before risk adjustment was 1.33, with p value=0.805. When this odds ratio was adjusted with POSSUM mortality scores, the odds of mortality reduced to 0.620 and the p value was 0.711. This drop in odds ratio for mortality was not significant. There was significant association of POSSUM morbidity and mortality scores on moving from subgroup B to subgroup A. Thus, there were 10 times more chances for getting higher morbidity score in subgroup A patients as compared to subgroup B.

## Discussion

Laparotomy for perforation peritonitis is a commonly performed emergency surgery in tropical countries [4]. The aetiology of intestinal perforation varies from region to region because of differences in socioeconomic factors and prevalent diseases. Thus, enteric fever and ileal perforation as its complication is common in our country [5–7]. Our study supports that duodenal perforation is the commonest cause of peritonitis and enteric fever accounts for more than 80% of all ileal perforations. It further emerged that EP is the disease of young males in our set up, the reason for which is unclear.

Enteric fever is reported to be associated with bowel complications in later stages (3^rd^ or 4^th^ week); however, we found that EP patients presented with fever of short duration (<1week). This may be due to greater virulence of the causative organism. Other studies have also reported shorter duration of symptoms with perforation. Once again, classically, patients of enteric fever are reported to present with step-ladder pattern of fever. We did not find so. There may be various factors responsible for this, the most important being pre-hospitalization treatment with antibiotics and antipyretics. The clinician therefore, should not entirely depend upon the classical history to diagnose enteric fever and perforation.

Traditionally, the diagnosis of enteric fever can only be confirmed by isolation of the Salmonella bacteria from blood, or some other body fluid, such as urine, stool or bone-marrow aspirate. But isolation rate is very low from various cultures [8–10]. In the present study salmonella could not be isolated by standard culture technique in any of the patients with Ileal perforation, most probably due to rampant use of antibiotics during pre-hospital treatment. In our study, we therefore relied on the histopathological (HP) confirmation of enteric aetiology of the ileal perforation. We calculated sensitivity and specificity of two widely used serological tests – Widal and Typhidot against HP positive cases with clinical suspicion [11–13]. Sensitivity and specificity of Widal was comparable with other studies in this region. Sensitivity and specificity of Typhidot was lower as compared to other studies. These authors had done their study in patients of typhoid fever without having any complication [14–16].

Relationship between duration of peritonitis and organ failure at admission has not been studied earlier. We felt that this was an important aspect of peritonitis, and found direct relationship between mean duration of peritonitis with different complications at presentation. With increasing duration of peritonitis chances of complications like dyselectrolytemia, ARDS, ARF and MODS increased in the order listed. However, shock showed inverse or no relation to duration of peritonitis. It is difficult to explain the significance and reason for this observation; it may be due to adverse effect of duration of peritonitis on outcome.

We could broadly categorize ileal perforation patients in to two: those with EP and those with NSIP. It was of interest to see if we could differentiate these on clinical grounds. Therefore, several factors were studied; it emerged that BMI, SIRS and hospital stay could differentiate the two. We found no study that has attempted this differentiation. A lower BMI in NSIP may be due to tuberculosis as the underlying cause in most of these cases. A higher SIRS in EP patients is possibly due to systemic nature of enteric infection. For similar reason, EP patients experienced complications more often than NSIP patients. Finally, mean hospital stay was significantly longer in EP patients. This is, possibly, due to comparatively higher morbidity in these patients.

POSSUM has proved to be accurate tools for audit of results of emergency surgery in our country [17, 18]. However, its usefulness as audit tool exclusively in EP patients has not been examined earlier although it occurs in almost epidemic proportions in our country. We found that POSSUM accurately predicted morbidity but not the mortality. Ileal perforations can be dealt with a number of operative procedures [19–21]. Commonly performed operative procedures include exteriorization of the bowel at the site of perforation, repair of the perforation and proximal ileostomy, primary repair of the perforated site and resection-anastomosis of the perforated segment. In this series, ileostomy was the most common operative procedure done in all ileal perforation patients. It was carried out in 40 (75.47%) patients. Other operative procedures performed were primary repair of perforation (12/22.64%) and resection/anastomosis (1/1.9%). Using crude mortality rates we found that there was no mortality in EP patients undergoing primary repair while mortality was 10% in ileostomy patients. This difference was also seen in all Ileal perforations (EP+NSIP) patients, with mortality of 10% in ileostomy group as compared to 7.5% in primary repair group. These observations suggest that patients undergoing ileostomy for either EP or NSIP origin tend to have poorer outcome [22–24]. Similar finding have been noted by Altamanap et al. This gives us an impression that ileostomy is a bad surgical choice. Mortality in ileostomy patients may be high from several factors, the most important of which is (perhaps) the fact that ileostomy is preferred in patients with poor general condition. This is supported by the higher POSSUM and P-POSSUM scores in these patients in our series. Therefore, when the risk is adjusted by applying the POSSUM/P-POSSUM scores the odds of mortality from ileostomy reduce considerably [25–27]. This has been previously demonstrated by Mohil et al. [28]. These observations suggest that to draw any conclusions regarding mortality and morbidity crude rates should not be relied upon. Further, ileostomy as the operative procedure should not be criticized to have higher mortality [29–32]. EP patients are generally very sick patients. Their postoperative hospital course is punctuated by significant morbidity. This is proved by our observations (morbidity rate- 79%) as well as by a recent study from Safdarjang hospital (morbidity rate- 79.3%). However, morbidity in our study as well as in Safdarjang hospital study was substantially higher compared to the observations made by Edino et al (morbidity rate- 49.1%).

Surgical site infection (SSI) was the most common complication, occurring in 60.8% of EP patients. The SSI rate observed by us was slightly higher than most other studies, viz Atamanalp et al [33]: 55%; Mohil et al: 42%; Eldino et al [34]: 53.8%). Incidence of burst abdomen in our EP patients was similar (20%) to that found by other authors (Atamanalp et al: 33.3%; Mohil et al: 20%; Eldino et al: 25.1%). Overall, morbidity rate was comparable in both, EP and NSIP categories of patients [35]. Further, rate of various complications was also comparable in EP and NSIP patients.

## Conclusion

POSSUM is an accurate tool for predicting morbidity in EP patients. P-POSSUM is an accurate tool for predicting mortality in EP patients. Ileostomy is a good surgical option in patients presenting with EP. It is not associated with higher mortality and morbidity as is commonly believed. Risk adjustment with POSSUM and or P-POSSUM corrects the anomaly.
